# Convalescence Amid Trees and Flowers

**Published:** 1919-07-26

**Authors:** 


### HOSPITAL FOR W.A.A.C.S.

### Convalescence Amid Trees and Flowers.

(From the *Times*, July 17.)

During the war members of the Q.M.A.A.C., when they fell ill oversea and had to be sent home, found hospitality in the wards of the Endell Street Military Hospital. But this was only a temporary solution for treating casualties which were bound to occur in a large oversea *personnel* living under difficult conditions and without home comforts. It was obviously better that the women should have their own hospital, if possible, with a garden. The difficulty was to find a suitable hospital for women who had borne many of the same hardships as the oversea men.

Now they have their own hospital—a delightful house of pleasant rooms, gardens, and fine old trees, originally built by Mr. Pears, of toilet-soap fame, at Isleworth, within sound of the tramways, but not within sight of them. When it was offered to the War Office as a hospital for mental cases it was found unsuitable, and as its defects for one purpose were its qualities for another, the director of the Q.M.A.A.C. medical department went down to see it, and found it exactly what she wanted. The first sick W.A.A.C.s went there in January during the influenza epidemic, and ever since its pleasant wards have been filled with medical and surgical cases from oversea.

The other day the Controller-in-Chief of the Q.M.A.A.C., Dame Florence Leach, went down to Isleworth to see her sick W.A.A.C.s, accompanied by her chief medical officer. The comfortable casual looking house is deceptive in its appearance, and a thorough "inspection" is a matter of a couple of hours. The cases that are not stretcher ones are taken to pleasant top rooms. Small friendly wards have three beds, and others more according to their size, and the higher one went in the house the healthier seemed the cheeks and the brighter the hair-ribbons of the girls, all of whom were medical cases of a minor kind. Lower down one came on surgical wards where an appendix or tonsils had been removed, and in another a girl, who had met with an accident in Bourges, is convalescing after the removal of a leg. She is able to move about on crutches, and was the privileged jester of the ward.

There is an operating theatre, shining and gleaming with its surgeons' paraphernalia, and a dispensary that has the usual wealth of bottles and mingled odours. Also rambling overhead, as if it had been specially built for the purpose, is a little flat where the medical staff live. There are three doctors on the permanent staff. The large ground floor wards are for the more serious cases. There are four of them, and a small one for sick W.A.A.C. officers. Beautiful gracious rooms, with wide windows, and doors opening on to the terrace, and everywhere with a pleasant vista of trees and flowers. There is a great court opening in the grounds, with a fountain crowned with a Cupid riding a dolphin, and here there is a gramophone given by the Queen to amuse the convalescent W.A.A.C.s, some of whom sat around listening to it, and sewing or reading. There is a charming winter garden for them, with plenty of ferns, and opening off it there is another of the unexpected extra rooms which can be used in case of necessity as an isolation ward.

Even this does not exhaust all the living space, and the homelike staff of fifty-seven W.A.A.C.s has commodious quarters in the garage stable *annexe*, where the lofts, unusually lightsome and airy, have been adapted for an ideal recreation room. The basement of the house proper is another source of pride to the C.O. There, entered by wide stairs, are corridors of sunny rooms, some of which contain surplus stores of many kinds, and others act as dining-rooms for the staff, and kitchens. There are several gardens, but there is only one gardener, and how she keeps her domain with its fierce delight in every kind of growth, from weeds to vegetable marrows, in order is a puzzle to the ordinary visitor. But its rich greens and its pleasant air of welcome make it a homely place to come when one is ill and then grow well in. And all the patients have that air of leisured happiness which suggests peace after the storm and stress of their work overseas.

Offers of occasional loans of motor-cars would be much appreciated by the matron for her charges who are having a long convalescence. Those who drive about every day sometimes do not realise the charm to girls who have been long in one place, be it ever so pleasant, of a swift view of something different. It gives them something new to talk about.

